# Gender Perspectives in Intimate Partner Violence: The Influence of Alexithymia in Peri-Urban Contexts

**DOI:** 10.3390/healthcare13080853

**Published:** 2025-04-09

**Authors:** Alejandra Coronel-Dávila, Georgina Zavaleta-Aguilar, Carlos Pérez-Lara

**Affiliations:** School of Psychology, Faculty of Health Sciences, Trujillo Campus, Universidad Cesar Vallejo, Las Magnolias 491, Victor Larco Herrera, Trujillo 13009, Peru; acoronelda@ucvvirtual.edu.pe (A.C.-D.); cperezla@ucvvirtual.edu.pe (C.P.-L.)

**Keywords:** intimate partner violence, alexithymia, gender, peri-urban contexts, emotional influences

## Abstract

**Background/Objectives**: Intimate partner violence (IPV) remains a critical social issue, with gender perspectives offering valuable insights into its dynamics. Recent studies suggest that alexithymia, or the inability to identify and express emotions, may play a significant role in exacerbating IPV, especially in peri-urban areas. The general objective of this study was to determine the moderating role of gender in the relationship between alexithymia and intimate partner violence among adults in populated centers in Trujillo in 2024. **Methods**: A correlational research design with explanatory scope was employed, including moderation analysis. The sample consisted of 108 adults aged 18 to 35 from populated centers. The instruments used included the Intimate Partner Violence Scale, the Toronto Alexithymia Scale, and a sociodemographic questionnaire. **Results**: Results indicated that, in the alexithymia variable, females predominated with a high level (78%), followed by a medium level (19%). In the intimate partner violence variable, females also predominated, with 48% at a high level and 39% at a medium level. Additionally, a positive, moderate-to-high, and significant correlation (ρ = 0.78, *p* < 0.001) was found between alexithymia and intimate partner violence. **Conclusions**: The study concluded that gender does indeed serve as a moderating variable (E = 1.42, *p* < 0.001) in the relationship between alexithymia and intimate partner violence in adults from populated centers.

## 1. Introduction

An individual may exhibit distinct and significant characteristics influenced by their gender, making it crucial to understand these differences within diverse sociocultural and socioemotional contexts [1]. Violence is a phenomenon that can occur across various circumstances, irrespective of social class. It often remains underrecognized when normalized. Some manifestations of violence are particularly evident within family environments, commonly referred to as domestic violence [2]. Accordingly, the present study is aligned with one of the Sustainable Development Goals (SDGs), specifically Goal 3: Good Health and Well-being, which aims to reduce premature mortality from non-communicable diseases by one-third through prevention and treatment, while promoting mental health and overall well-being [3].

Victims of domestic violence often endure mild-to-severe physical aggression, verbal abuse, psychological harm, and other forms of mistreatment [4], and these experiences can have significant repercussions on individuals’ holistic and developmental well-being [5], including a heightened likelihood of adverse impacts on their ability to recognize and express emotions.

In Asia, a study involving 1466 Japanese men who reported experiencing violence in their intimate relationships found that approximately 19% sought formal support, 65% relied on informal support, and 16% utilized both [6]. In England, slightly over 20% of 7058 adults reported being victims of intimate partner violence [7]. In North America, nearly 50% of women and slightly more than 40% of men have been victims of intimate partner violence in the United States [8]. In South America, it was found that, among 3860 cases of domestic violence against Colombian women, 65% were perpetrated by their partners [9]. Similarly, in another South American country, a study revealed a higher prevalence of psychological violence, with 78% of the victims being women. The sample consisted of 50 Peruvian adults, including 25 women and 25 men, aged between 20 and 40 years [10].

On the other hand, alexithymia is understood as a cognitive difficulty in which individuals struggle to identify and interpret sensations related to emotions, potentially limiting their social skills [11]. Furthermore, individuals with this condition are often characterized as avoidant and may exhibit difficulties empathizing or understanding others’ perspectives [12]. Moreover, according to models such as the sociocultural, psychodynamic, and neuropsychological frameworks, alexithymia may be associated with the development of various mental health problems [13].

The moderating role of gender is conceptualized as the roles and responsibilities defined for men and women by society and culture, which are assigned from childhood [14]. Regarding gender identity, it is described as an emotional and cognitive–behavioral conviction, whereas sex is defined by one’s biological genitalia at birth. Although these concepts are distinct, they are interrelated; gender is often determined by biological sex, whether the identity aligns or diverges from it [15]. Based on this, it is observed that females exhibit significantly higher symptoms of depression, anxiety, social anxiety, and emotional regulation difficulties compared to males. These disparities result in notable internalizing and externalizing problems [16]. Such findings highlight the psychological differences between genders, which may have important clinical implications for the prevention and treatment of emotional disorders [17].

In Peru, a “populated center” refers to communities with access to basic services, including primary or secondary education, and comprising more than 150 individuals residing in clustered or partially dispersed households [18]. Similarly, concerning violence and its relationship to gender in the context of Peruvian populated centers, it is common for women to be the most affected [19] as such violence is often used as a means of control to maintain them in a position of inferiority [20].

It is important to note that Peru has a large number of populated centers, and not all have access to the services of a Women’s Emergency Center (WEC). These centers are prioritized for coverage in provinces and districts with the highest rates of violence. However, despite an increase in the number of WECs, challenges remain in their distribution and accessibility, particularly in rural and hard-to-reach areas [21].

Regarding the relationship between alexithymia and intimate partner violence, research has shown that low income levels and exposure to emotional violence significantly increase both the acceptance of violence and alexithymic tendencies [22]. Additionally, a positive relationship has been observed between alexithymia and impulsivity, alexithymia and anger, and impulsivity and anger. This suggests that individuals with alexithymia are more likely to commit crimes outside the family environment [23]. Similarly, the presence of parental indifference, maternal indifference, and paternal physical coercion has been strongly correlated with higher levels of alexithymia [24]. On the other hand, studies have found no significant contribution of alexithymia to other known correlates of suicide and violence [25]; furthermore, no association has been identified between alexithymia, violent behavior, and criminality [26].

Concerning alexithymia and gender, research has identified intimate partner violence as a significant risk factor for alexithymia [27]; moreover, findings indicate that males exhibit a higher prevalence of alexithymia compared to females [28].

Regarding gender and intimate partner violence, research has concluded that men tend to remain in abusive relationships with high levels of partner violence for longer periods than women [29]. Conversely, women exhibit a higher prevalence of victimization in cases of intimate partner violence [30], with such situations occurring more frequently among young adult women [31].

The psychological framework underpinning this study is the ecological model proposed by Bronfenbrenner (1987), which explains how the various social contexts in which individuals interact directly impact their moral, cognitive, and relational development [32], and this framework is particularly relevant in understanding alexithymia. Furthermore, the origins of domestic violence, based on systemic influences, highlight the primary social circle’s significant role in shaping individuals [33]. At the family level, shared beliefs and ideologies often sustain violent behavioral patterns, perpetuating a repetitive cycle of violence [34]. The ecological model introduces a hierarchy of levels based on the systems interacting with the individual [33]. These levels include the following: microsystems, the individual and their immediate environment; mesosystems, closer social circles such as family or a partner; ecosystems, broader social contexts that are not directly immediate; and macrosystems, societal perceptions and ideologies [35].

The present research is theoretically justified as it expands knowledge regarding the moderating role of gender in the relationship between alexithymia and intimate partner violence among adults in populated centers. Similarly, it is practically justified as it provides innovative data and information to future researchers or readers on whether gender serves as a moderating factor in alexithymia and intimate partner violence. Additionally, the findings can contribute to the design of appropriate or updated research plans for future studies. From a methodological standpoint, the study offers updated statistical data using tools that provide evidence of validity and reliability, such as the Toronto Alexithymia Scale (TAS-20) and the Family Violence Measurement Scale (VIFJ4). These tools will enable the determination and identification of the moderating role of gender among adults in populated centers. Socially, this research gathers updated data on alexithymia and intimate partner violence, focusing on data collection in populated centers at a higher risk of a relationship between these two variables. The findings include statistical data for comparison and allow the identification of the gender most affected by this cause-and-effect relationship.

The research posed the following question: Does gender act as a moderating role in the relationship between alexithymia and intimate partner violence among adults in populated centers? The general objective was to determine the moderating role of gender in the relationship between alexithymia and intimate partner violence among adults in populated centers in Trujillo in 2024. The specific objectives were as follows: (O1) to establish a demographic data table based on gender; (O2) to identify the level of alexithymia among adults in populated centers in Trujillo in 2024; (O3) to identify the level of intimate partner violence among adults in populated centers in Trujillo in 2024; (O4) to determine whether there is a significant relationship between alexithymia and intimate partner violence, including its dimensions, in Trujillo in 2024; and (O5) to determine whether there are significant differences in intimate partner violence according to gender among adults in populated centers in Trujillo in 2024.

## 2. Method

### 2.1. Design

This study adopts a quantitative approach, as it relies on a series of rigorous procedures organized sequentially to test hypotheses derived from a theoretical perspective. This approach is characterized by the methodological use of techniques associated with measuring the units of analysis, which is supported by statistical tools and the use of simples [36,37]. Additionally, the research design is correlational, as it facilitates the measurement of the study variables, domestic violence and alexithymia, and the analysis of their relationship. First, each variable is independently evaluated, described, and quantified, and their association is analyzed to later understand their joint behavior [36]; furthermore, the study has an explanatory scope, aiming to comprehend and describe the variables under analysis [38].

### 2.2. Participants

The sample consisted of 108 adults residing in rural areas of Trujillo, specifically in the districts of El Milagro and Huanchaco. Inclusion criteria included adults aged 18 to 35 years, both men and women, and residents of rural areas. Exclusion criteria encompassed individuals currently undergoing psychiatric treatment to minimize the risk of vulnerability [39], those who opted out of the study, individuals who did not complete the instruments, and adults who did not provide informed consent. The sample size was calculated based on the following parameters: effect size *f*^2^ = 0.15, alpha error probability *a* = 0.05, statistical power (1—beta error probability) = 0.95, and two predictors. Calculations were performed using G*Power software version 3 [40], aiming to minimize type I and type II errors in the interpretation of the results [41].

### 2.3. Instruments

#### 2.3.1. Domestic Violence Scale (VIFJ4)

The Domestic Violence Scale (VIFJ4), developed by Dr. Julio Jaramillo in 2014, measures the type and severity of domestic violence. It is designed for individuals aged 18 to 35 and consists of 25 items rated on a Likert-type scale: “almost always” (5), “often” (4), “sometimes” (3), “rarely” (2), “almost never” (1), and “never” (0). The variable domestic violence is divided into the following dimensions: gender violence (25, 24, 23), patrimonial violence (22, 21, 20), social violence (19, 18, 17, 16), sexual violence (15, 14, 13, 12, 11, 10), psychological violence (9, 8, 7, 6, 5), and physical violence (4, 3, 2, 1). Scoring is assigned based on responses as follows: “never” = 0, “almost never” = 1, “rarely” = 2, “sometimes” = 3, “often” = 4, and “almost always” = 5, where 0 indicates no violence, scores up to 35 indicate mild violence, scores from 36 to 67 reflect moderate violence, and scores of 68 or above suggest severe violence. In this study, the reliability of the scale was assessed using the internal consistency method, yielding a satisfactory Cronbach’s alpha coefficient (α = 0.96).

#### 2.3.2. Toronto Alexithymia Scale (TAS-20)

The Toronto Alexithymia Scale (TAS-20), originally developed by Bagby, Parker, and Taylor (1994), was adapted into Spanish by Dr. Francisco Martínez Sánchez (1996). This instrument aims to measure alexithymia in adult populations aged 18 and older. It consists of 17 items rated on a Likert-type scale: 1 = “strongly disagree”, 2 = “moderately disagree”, 3 = “neither agree nor disagree”, 4 = “moderately agree”, and 5 = “strongly agree”. This instrument focused on difficulty identifying emotions (14, 13, 9, 7, 6, 3, 1), difficulty verbally expressing emotions (17, 12, 11, 4, 2), and Externally Oriented Thinking (20, 19, 18, 16, 15, 10, 8, 5). The total score is calculated by summing the item responses on a scale from 1 to 5, with items 4, 5, 10, 18, and 19 being reverse-scored. Scores below 61 indicate low alexithymia, while scores of 61 or higher suggest high alexithymia. In this study, the reliability of the scale was assessed using the internal consistency method, yielding a satisfactory Cronbach’s alpha coefficient (α = 0.74).

#### 2.3.3. Sociodemographic Form

The sociodemographic form collects data on various participant characteristics, including age, department, province, district, gender, marital status, educational level, religion, and prior psychological treatment [42].

### 2.4. Procedure

Initially, authorization was obtained from 108 adults residing in rural areas to participate in the study. Participants were then provided with the corresponding questionnaires on intimate partner domestic violence and alexithymia, and, lastly, they completed the sociodemographic form. The entire process took approximately 45 min per participant.

The collected data were entered into an Excel database for descriptive analysis, which included calculating the mean and standard deviation. Subsequently, the distribution of variables was evaluated using the Kolmogorov–Smirnov normality test with Lilliefors significance correction and the Mann–Whitney U test to assess gender heterogeneity. Inferential analysis was conducted using variable correlation tests in the SPSS Statistics 27 software, while moderation effects were analyzed using the Jamovi 2.3.28 software. The results were presented in statistical tables.

### 2.5. Statistical Analysis

Data were obtained through surveys designed for each variable and its corresponding dimensions. Responses were entered into a database and organized using frequency tables. Additionally, measures of central tendency such as mean and median, standard deviation, and normality tests were applied, specifically the Kolmogorov–Smirnov test with Lilliefors correction, and the Mann–Whitney U test. Since the variables did not show a normal distribution, Spearman’s Rho test was employed using the SPSS software. Finally, a moderation analysis was performed using the Jamovi software.

### 2.6. Ethical Considerations

This study was conducted respecting the principles of freedom and self-determination of the participants, as well as ensuring non-discrimination to uphold equality. The identity of the participants was protected through anonymity. Additionally, the results obtained reflect reality without alterations, in accordance with the Declaration of Helsinki by the World Medical Association [43], the guidelines of the American Psychological Association, seventh edition [44], and the approval of the university’s ethics committee under code 0470-2022.

## 3. Results

In Table 1, which details the distribution of sociodemographic data, it is observed that, among males, religious practice is predominant, accounting for 69% (*n* = 37). Similarly, in terms of marital status, married individuals represent the majority at 54% (*n* = 29). The most common educational level in this group is higher education completion, accounting for 33% (*n* = 18). Regarding age, the predominant group comprises adults aged 18 to 23 years, accounting for 39% (*n* = 21).

On the other hand, among females, religious practice is also predominant, accounting for 72% (*n* = 39). Regarding marital status, cohabiting individuals are the majority, accounting for 67% (*n* = 36). In terms of educational attainment, the most common level is complete secondary education, representing 33% (*n* = 18). Finally, the predominant age group among females corresponds to adults aged 24 to 29 years, accounting for 39% (*n* = 21).

In Table 2, regarding the distribution and frequency of the alexithymia variable, it can be observed that, for the male gender, in the dimension of Difficulty Identifying Feelings (DIS), the most common level is low, with 59% (*n* = 32). Similarly, in the dimension of Difficulty Describing Feelings (DDS), the low level also predominates with 72% (*n* = 39). Additionally, in the dimension of Externally Oriented Thinking (POE), the low level is predominant with 67% (*n* = 36).

On the other hand, for the female gender, in the dimension of Difficulty Identifying Feelings (DIS), the most common level is medium, with 63% (*n* = 34). In the dimension of Difficulty Describing Feelings (DDS), the medium level predominates with 81% (*n* = 44). Furthermore, in the dimension of Externally Oriented Thinking (POE), the medium level predominates with 74% (*n* = 40).

In Table 3, concerning the distribution and frequency of the variable intimate partner domestic violence, it is observed that, among males, a low level of violence predominates, accounting for 98% (*n* = 53). This result includes the dimensions of physical violence (VF), psychological violence (VPS), sexual violence (VSX), social violence (VSC), patrimonial violence (VPT), and gender-based violence (VG).

In contrast, among females, a high level of violence predominates, with 48% (*n* = 26), particularly in the dimensions of sexual violence (VSX) and gender-based violence (VG).

In Table 4, regarding the male gender, it is observed that the variables alexithymia and intimate partner domestic violence, as well as the dimensions Difficulty Identifying Feelings, Difficulty Describing Feelings, Externally Oriented Thinking, physical violence, psychological violence, sexual violence, social violence, patrimonial violence, and gender-based violence, do not exhibit a normal distribution (*p* < 0.05).

On the other hand, for the female gender, the variables alexithymia and the dimensions Difficulty Identifying Feelings, Difficulty Describing Feelings, physical violence, psychological violence, sexual violence, and patrimonial violence also do not exhibit a normal distribution (*p* < 0.05).

In Table 5, it is evident that, for the variable alexithymia, the mean for men (M = 44.7, *p* < 0.01) is significantly lower than the mean for women (M = 66.9, *p* < 0.01), with a Mann–Whitney U value (U = 256.00) and statistical significance (*p* < 0.01).

Regarding the variable intimate partner domestic violence, the difference between the mean for women (M = 65.1) and the mean for men (M = 13.2) is also statistically significant (U = 119.50, *p* < 0.01).

In Table 6, it is shown that the variable alexithymia has a high, significant positive correlation (ρ = 0.88, *p* < 0.001) with the variable intimate partner violence. The following correlations with its dimensions are also observed: physical violence shows a moderate, significant positive correlation (ρ = 0.65, *p* < 0.001); psychological violence presents a high, significant positive correlation (ρ = 0.73, *p* < 0.001); sexual violence displays a moderate, significant positive correlation (ρ = 0.65, *p* < 0.001); social violence exhibits a high, significant positive correlation (ρ = 0.71, *p* < 0.001); property violence presents a moderate, significant positive correlation (ρ = 0.43, *p* < 0.001); and finally, gender-based violence shows a moderate, significant positive correlation (ρ = 0.68, *p* < 0.001).

In Table 7, it is evident that, in relation to intimate partner violence, the presence of a high level of alexithymia significantly increases (*p* < 0.001) the likelihood of experiencing higher levels of intimate partner violence. Similarly, gender has a significant influence on the likelihood of experiencing intimate partner violence (*p* < 0.001). Furthermore, when the variables alexithymia and gender are present in the same individual, the probability of suffering from intimate partner violence increases significantly (*p* < 0.001).

On the other hand, concerning the moderating variable, belonging to the male gender does not have a significant impact (*p* > 0.05) on the likelihood of being a victim of intimate partner violence, while belonging to the female gender has a significant impact (*p* < 0.001) on the probability of being a victim of intimate partner violence.

Moreover, regarding the levels of alexithymia, having a high or average level significantly increases (*p* < 0.001) the probability of experiencing intimate partner violence, whereas exhibiting a low level significantly reduces (*p* > 0.05) the likelihood of being a victim of intimate partner violence.

## 4. Discussion

Regarding the general objective, which was to determine the moderating role of gender in the relationship between alexithymia and intimate partner violence (IPV) among adults in populated centers, the results indicate that IPV significantly increases (*p* < 0.001) in the presence of high levels of alexithymia. Additionally, gender exerts an influence on the presence of IPV (*p* < 0.001). This suggests that the variable gender does indeed serve as a moderator between alexithymia and IPV in adults from populated centers (see Table 7). In a study conducted with a sample of 140 women over 18 years of age, it was found that exposure to violence in the current relationship increased acceptance of violence, while long-term resentment toward the partner heightened alexithymic attitudes [22]. Concerning the variable of violence, it can be considered one of the social phenomena present across all areas of human life. Similarly, violence within the family and gender-based violence remain among the most serious public health issues on a global scale [45]. The findings of the present research support the results reported [22], as it was determined that gender plays a moderating role in the relationship between alexithymia and intimate partner violence (IPV). This may be explained by violations of norms governing family coexistence, particularly within the couple dynamic. Such transgressions are more likely to affect individuals with difficulties in expressing and understanding emotions, whether interpersonally or intrapersonally, and are influenced by the social roles associated with gender.

The first specific objective was to establish a sociodemographic data table for the moderating variable gender. According to the results, in the male group, the majority are religious practitioners (69%), married adults (54%), individuals with completed higher education (33%), and those aged 18 to 23 years (39%). In contrast, in the female group, a higher percentage of religious practitioners was observed (72%), with cohabitation as the predominant marital status (67%). Regarding the education level, the most prominent category was completed secondary education (33%), and the dominant age range was 24 to 29 years (39%) (see Table 1). In a study conducted on a Spanish population aged 18 years and older, it was found that 62.8% of women were married, 30.4% had two children, 40.2% had completed primary education, and their primary occupation was homemaking [30]. Additionally, a sociodemographic study was conducted with women aged 15 to 49 years. The results indicated that 57.3% were young adults, 58.7% lived in urban areas, and the predominant marital status was single (38.7%), followed by married women (32.2%). Among the married women, 39% reported homemaking as their primary occupation, and 37.9% had completed secondary education [31]. The sociodemographic profile facilitates the collection of essential information or data that support the development of research [42]. The data obtained indicate that there is no clear predominance in marital status within the female gender. However, comparisons with previous studies reveal similarities in categories such as the educational level, occupation, and age of the participants, as well as in religious affiliation and practice.

Regarding the second specific objective, which was to identify the level of alexithymia among adults in populated centers, the results revealed that male adults predominantly exhibited low levels of alexithymia (74%), whereas female adults showed high levels of alexithymia (78%) (see Table 2). This indicates that alexithymia is more prevalent among females (U = 256.00, *p* < 0.05) (see Table 5). In a related study conducted with a sample of 56 university students (21 males; 35 females), it was found that 77% of females and 85% of males presented alexithymia [28]. Alexithymia is an emotional awareness disorder characterized by difficulties in differentiating, regulating, and responding to emotions in humans [46]. The data obtained in the present research allow us to reject the results presented earlier [28]. The results of our study indicate that females in the context of violence experience greater disturbances in emotional awareness.

Furthermore, regarding the third specific objective, which was to identify the level of intimate partner violence (IPV) in adults from populated centers, the results revealed that male adults predominantly exhibit low levels of IPV (98%), whereas female adults show a higher prevalence of high levels of IPV (48%) (see Table 3). This suggests that females are more likely to experience intimate partner violence (U = 119.50, *p* < 0.05) (see Table 5). In a study conducted with a population of women from the Azogues canton, it was found that more than 50% of the adult population reported experiencing various forms of violence, including property violence (22.5%), psychological violence (45.6%), physical violence (20.8%), and sexual violence (13.1%) [31]. No previous studies have been found that analyzed intimate partner violence in male populations. Intimate partner violence is considered any act of sexual, psychological, or physical abuse perpetrated by one member of the partner circle against the other [47]. The data obtained in the present research support the results presented, as it was found that intimate partner violence is more prevalent among females. This is due to societal norms and hierarchical power differences based on gender, which place adults in varying situations of risk, such as violence [31].

Similarly, in the fourth specific objective, which was to determine whether there is a significant relationship between alexithymia and intimate partner violence and its dimensions, the results show that the alexithymia variable has a strong, positive, and significant correlation (r = 0.88 ***, *p* < 0.001) with the intimate partner violence variable and its dimensions: psychological violence (r = 0.73 ***, *p* < 0.001), social violence (r = 0.71 ***, *p* < 0.001), and gender-based violence (r = 0.68 ***, *p* < 0.001) (see Table 6). This suggests that a higher presence of alexithymia is associated with a greater likelihood of experiencing intimate partner violence. Additionally, in a study conducted with 40 women who were victims of intimate partner violence and 40 women from a control group, it was found that alexithymia was highly correlated with partner violence (r = 0.633, *p* < 0.001) [27]. Alexithymia is a risk factor due to the challenges it presents in intra- and interpersonal emotional interactions [48]. The data obtained in the present research support the mentioned results, due to the evidence that highlights the relationship between alexithymia and intimate partner violence [27], since alexithymia levels would affect emotional responses, particularly in violent situations within the couple.

In the fifth specific objective, which was to determine whether there is a significant difference in intimate partner violence based on gender in adults from populated centers, the results showed that being male does not have a significant impact (*p* > 0.05) on the likelihood of being a victim of intimate partner violence. In contrast, being female has a significant impact (*p* < 0.001) on the likelihood of being a victim of intimate partner violence, indicating a significant difference, with females being more affected (see Table 7). A study conducted with 2060 adults, where 62.1% were female and 37.9% were male, concluded that males experience higher rates of intimate partner violence compared to females [29]. Any act that causes harm to a partner is a form of violence, with the most common types being physical and psychological behaviors exhibited by the intimate partner. Violence tends to occur more frequently against women [49]. The data obtained in the present research allow us to reject the previous results [29], given that it was apparent that the female gender demonstrates a significant difference in intimate partner violence.

### 4.1. Limitations

One of the limitations of the present study is the type of sampling, which was non-probabilistic and basic in nature. This sampling method is primarily used for research aimed at expanding and obtaining new knowledge regarding a particular phenomenon or event. As a result, it limited the generalization of the findings, meaning that the external validity is confined to the participants in this study. Future researchers may consider expanding the scope of the study variables by increasing the sample size and exploring different cultures or traditions. Additionally, a limitation was observed in the limited number of studies that address the variables in the male population.

### 4.2. Implications

The findings of this study have theoretical implications in that they contribute to expanding knowledge about the moderating role of gender in the relationship between alexithymia and intimate partner violence among adults in rural areas. Specifically, they demonstrate that alexithymia is not only related to intimate partner violence but that its impact may also vary according to gender. In practical terms, these results provide innovative or updated data for future researchers. Clinically, health professionals can adapt treatment programs or plans, thereby enhancing the effectiveness of their interventions, with a personalized approach based on gender and socioemotional needs. Furthermore, from a social perspective, the study collected updated data on alexithymia and intimate partner violence in rural areas, examining how individuals experience or react differently to the proposed variables. This information can assist in the design of more inclusive policies tailored to the needs of each gender.

## 5. Conclusions

It is concluded that gender does indeed play a moderating role (estimate = 1.42, *p* < 0.001) in the relationship between alexithymia and intimate partner violence among adults in rural areas. Additionally, there is a significant difference in intimate partner violence based on gender, where the probability of being a victim of intimate partner violence differs between males (*p* > 0.05) and females (*p* < 0.001).

## Figures and Tables

**Table 1:** Distribution of sociodemographic data.

		Male		Female		Total	
Religion		*n*	%	*n*	%	*n*	%
	Practicing	37	69	39	72	76	70
	Non-practicing	17	31	15	28	32	30
Marital status							
	Married	29	54	18	33	47	44
	Cohabiting	25	46	36	67	61	56
Educational level							
	Completed higher education	18	33	17	31	35	32
	Incomplete higher education	16	30	3	6	19	18
	Technical education	11	20	10	19	21	19
	Completed secondary education	7	13	18	33	25	23
	Incomplete secondary education	2	4	5	9	7	6
	Primary education	0	0	1	2	1	1
Age							
	30–35	13	24	19	35	35	32
	24–29	20	37	21	39	41	38
	18–23	21	39	14	26	32	30

Note: *n* = 108.

**Table 2:** Distribution and frequency of the alexithymia variable by gender.

	Level	DIF		DDF		EOT		Total	
		*n*	%	*n*	%	*n*	%	*n*	%
Total	High	16	15	4	4	11	10	51	47
	Middle	55	51	58	54	57	53	15	14
	Low	37	34	46	43	40	37	42	39
Male	High	1	2	1	2	1	2	9	17
	Middle	21	39	14	26	17	31	5	9
	Low	32	59	39	72	36	67	40	74
Female	High	15	28	3	6	10	19	42	78
	Middle	34	63	44	81	40	74	10	19
	Low	5	9	7	13	4	7	2	4

Note: *n* = 108. DIF = Difficulty Identifying Feelings; DDF = Difficulty Describing Feelings; EOT = Externally Oriented Thinking.

**Table 3:** Distribution and frequency of intimate partner violence by gender.

	Level	PV		PSV		SXV		SCV		PTV		GV		Total	
		*n*	%	*n*	%	*n*	%	*n*	*%*	*n*	*%*	*n*	*%*	*n*	*%*
Total	High	6	6	1	1	25	23	3	3	4	4	30	28	26	24
	Middle	38	35	43	40	21	19	34	31	20	19	17	16	22	20
	Low	64	59	64	59	62	57	71	66	84	78	61	56	60	56
Male	High	0	0	0	0	0	0	0	0	0	0	0	0	0	0
	Middle	0	0	4	7	2	4	4	7	2	4	4	7	1	2
	Low	54	100	50	93	52	96	50	93	52	96	50	93	53	98
Female	High	6	11	1	2	25	46	3	6	4	7	30	56	26	48
	Middle	38	70	39	72	19	35	30	56	18	33	13	24	21	39
	Low	10	19	14	26	10	19	21	39	32	59	11	20	7	13

Note: *n* = 108. PV = physical violence; PSV = psychological violence; SXV = sexual violence; SCV = social violence; PTV = patrimonial violence; GV = gender-based violence.

**Table 4:** Descriptive statistics and normality test.

Variables and Dimensions		Male				Female				Total			
		M	SD	*K-S*	*p*	M	SD	*K-S*	*p*	M	SD	*K-S*	*p*
Alexithymia		44.7	12.1	0.15	0.00	66.9	9.6	0.12	0.03	55.8	15.5	0.11	0.00
	Difficulty Identifying Feelings	15.6	4.8	0.14	0.00	23.3	4.6	0.14	0.01	19.4	6.1	0.11	0.00
	Difficulty Describing Feelings	11.0	3.5	0.18	0.00	16.5	3.0	0.19	0.00	13.8	4.3	0.14	0.00
	Externally Oriented Thinking	18.1	6.0	0.19	0.00	27.0	5.2	0.09	0.20	22.6	7.1	0.11	0.00
Intimate partner violence		13.2	8.0	0.14	0.01	65.1	23.5	0.11	0.09	39.2	31.4	0.20	0.00
	Physical violence	1.3	1.6	0.26	0.00	10.7	4.9	0.19	0.00	6.0	6.0	0.20	0.00
	Psychological violence	4.1	3.3	0.17	0.00	13.3	6.0	0.15	0.00	8.7	6.7	0.13	0.00
	Sexual violence	2.0	2.6	0.23	0.00	14.9	7.2	0.14	0.01	8.5	8.4	0.20	0.00
	Social violence	2.9	2.6	0.20	0.00	9.1	4.7	0.11	0.06	6.0	4.9	0.13	0.00
	Patrimonial violence	1.1	1.9	0.29	0.00	5.1	4.3	0.13	0.02	3.1	3.9	0.23	0.00
	Gender-based violence	1.9	2.3	0.25	0.00	11.9	6.3	0.09	0.20	6.9	6.9	0.22	0.00

Note: *n* = 108 (male = 54; female = 54). M = mean; SD = standard deviation; *K-S* = Kolmogorov–Smirnov test with Lilliefors significance correction; *p* = significance.

**Table 5:** Mann–Whitney U test.

Variables and Dimensions		Male		Female		U	*p*
		M	SD	M	SD		
Alexithymia		44.7	12.1	66.9	9.6	256.00	0.00
	Difficulty Identifying Feelings	15.6	4.8	23.3	4.6	381.50	0.00
	Difficulty Describing Feelings	11.0	3.5	16.5	3.0	358.00	0.00
	Externally Oriented Thinking	18.1	6.0	27.0	5.2	411.50	0.00
Intimate partner violence		13.2	8.0	65.1	23.5	119.50	0.00
	Physical violence	1.3	1.6	10.7	4.9	237.00	0.00
	Psychological violence	4.1	3.3	13.3	6.0	263.00	0.00
	Sexual violence	2.0	2.6	14.9	7.2	211.50	0.00
	Social violence	2.9	2.6	9.1	4.7	397.00	0.00
	Patrimonial violence	1.1	1.9	5.1	4.3	643.50	0.00
	Gender-based violence	1.9	2.3	11.9	6.3	241.50	0.00

Note: *n* = 108 (male = 54; female = 54). M = mean; SD = standard deviation; U = U of Mann–Whitney test; *p* = significance.

**Table 6:** Correlation test between the variables alexithymia and intimate partner violence.

Variables and Dimensions		1	1.1	1.2	1.3	2	2.1	2.2	2.3	2.4	2.5	2.6
1. Alexithymia		—										
	1.1 Difficulty Identifying Feelings	0.88 ***	—									
	1.2 Difficulty Describing Feelings	0.88 ***	0.73 ***	—								
	1.3 Externally Oriented Thinking	0.90 ***	0.65 ***	0.72 ***	—							
2. Intimate partner violence		0.78 ***	0.69 ***	0.69 ***	0.70 ***	—						
	2.1 Physical violence	0.65 ***	0.57 ***	0.59 ***	0.54 ***	0.77 ***	—					
	2.2 Psychological violence	0.73 ***	0.63 ***	0.63 ***	0.68 ***	0.89 ***	0.73 ***	—				
	2.3 Sexual violence	0.65 ***	0.54 ***	0.62 ***	0.56 ***	0.88 ***	0.69 ***	0.78 ***	—			
	2.4 Social violence	0.71 ***	0.65 ***	0.64 ***	0.61 ***	0.81 ***	0.56 ***	0.62 ***	0.66 ***	—		
	2.5 Patrimonial violence	0.43 ***	0.41 ***	0.40 ***	0.36 ***	0.67 ***	0.43 ***	0.50 ***	0.54 ***	0.63 ***	—	
	2.6 Gender-based violence	0.68 ***	0.56 ***	0.57 ***	0.64 ***	0.77 ***	0.59 ***	0.68 ***	0.60 ***	0.59 ***	0.47 ***	—

Note: *n* = 108 (male = 54; female = 54). *** *p* < 0.001.

**Table 7:** Moderating effect of gender on the relationship between alexithymia and intimate partner violence.

Effect		Estimated	SE	95% Confidence Interval		*p*
				Lower	Upper	
Alexithymia		0.91	0.13	0.66	1.16	0.00
Gender		−47.37	15.14	−77.39	−17.36	0.00
Alexithymia ✻ Gender		1.42	0.25	0.92	1.92	0.00
Gender						
	Male	0.20	0.16	−0.11	0.51	0.20
	Female	1.62	0.20	1.23	2.01	0.00
Alexithymia						
	High (+1 SD)	1.62	0.20	1.30	1.97	0.00
	Average	0.91	0.11	0.70	1.13	0.00
	Low (−1 SD)	0.20	0.16	−0.11	0.51	0.21

Note: *n* = 108 (male = 54; female = 54). SE = Standard Error; Lower = Minimum Value; Upper = Maximum Value; *p* = significance.

## Data Availability

The data used in this research are available at the following DOI: [10.17605/OSF.IO/TXCYJ] (https://doi.org/10.17605/OSF.IO/TXCYJ).
